# Exploring Potential Predictors and Mediators of Marijuana Use Among US Women: A Structural Equation Modeling Approach

**DOI:** 10.5888/pcd23.250495

**Published:** 2026-09-03

**Authors:** Elfreda Samman, Wah Wah Myint, Gabrielle Torres-Coley, Aishatu Yusuf

**Affiliations:** 1 College of Public Health, University of North Texas Health Science Center, Fort Worth, Texas; 2 School of Public Health, Center for Community Health and Aging, Texas A&M University, College Station, Texas; 3 School of Public Health, Department of Health Behavior, Texas A&M University, College Station, Texas

## Abstract

**Introduction:**

Despite concerns about the impact of marijuana on women’s health, studies exploring factors influencing use are limited. We examined predictors and mediators of marijuana use among US women.

**Methods:**

Structural equation modeling analyzed data on women (n = 16,081) from the 2023 National Survey on Drug Use and Health, with marijuana use as dependent variable. Sociodemographic variables and covariates included age, race and ethnicity, marital status, education, poverty status, county type, criminal history, and binge drinking. Potential mediators were past-year major depressive episode (PY–MDE); past-year use of illicit drugs, tobacco, and alcohol (PY–IDTA); poverty status; and body mass index.

**Results:**

Marijuana use was frequently reported among women aged 26 to 34 years (35.6%), those who were non-Hispanic White (60.8%), never been married (62.5%), with some college-level education (38.1%), living in large metropolitan areas (56.7%), those who did not binge drink (53.8%), without a criminal history (83.7%), with no PY–MDE (74.8%), no PY–IDTA (73.4%), those who were living above the poverty threshold (58.5%), and those with obesity (37.5%). Criminal history (β = 0.188) and binge drinking (β = 0.404) had positive direct effects on marijuana use; age (β **=**−0.065) and race (β **=** −0.027) had negative direct effects on use. PY–MDE mediated the relationships between age, race and ethnicity, marital status, county, past criminal history, binge drinking, and marijuana use (indirect βs **=** −0.008 to 0.080). PY–IDTA mediated the relationships between race and ethnicity, marital status, education, past criminal history, binge drinking, and marijuana use (indirect βs **=** −0.013 to 0.378), with all *P* values <.05.

**Conclusion:**

Results suggest potential predictors and mediators of marijuana use, highlighting the need for multi-strategy targeted interventions among US women.

**Summary**

**What is already known on this topic?**

Marijuana use is widespread among women in the US and is associated with mental, social, and reproductive health concerns and with other substance use issues.

**What is added by this report?**

We used data from the National Survey on Drug Use and Health and structural equation modeling to explore complex pathways of the potential predictors and mediators of marijuana use among US women aged 18 to 44 years. Marijuana use among US women was affected by an interplay of sociodemographic, psychological, and behavioral factors, with depression and other illicit drug use being key mediators.

**What are the implications for public health practice?**

Prevention efforts should move away from addressing single factors related to marijuana use and instead employ approaches using multiple strategies targeting factors related to marijuana use in women.

## Introduction

Marijuana use has become increasingly widespread, emerging as one of the most commonly used illicit drugs in the US (1). According to the 2023 National Survey on Drug Use and Health (NSDUH), 21.8% of people aged 12 years or older reported using marijuana in the past year. Adults aged 18 to 25 years had the highest percentage of those reporting use (36.5%), followed by adults aged 26 years or older (20.8%) (1), with the gender gap (more use seen among men) narrowing over time (2). Of concern is that marijuana use spans all demographic categories, including women aged 18 to 44 years.

Among women, marijuana use has been associated with a range of health concerns, including mental, social, and reproductive concerns as well as issues with substance abuse. Previous studies have also indicated that marijuana is linked to depression and poor mental health (3). Marijuana use was found to be associated with adverse neonatal outcomes such as an increased risk of preterm and low birth weight, small for gestational age (4,5), and negative effects on breastfeeding and future fertility (5). Additionally, cigarette use and binge drinking were found to be associated with marijuana use (3).

Prior studies identified several sociodemographic predictors of marijuana use, such as younger age, lower income, being unmarried, lower education, alcohol use, tobacco use, and use of other illicit substances (5). Understanding the underlying factors affecting marijuana use also requires exploring mediators that influence behavior.

Several factors that mediate the relationship between sociodemographic factors and marijuana use have been identified in previous studies: use of alcohol, tobacco, and illicit substances other than marijuana; psychological distress; perceived and peer norms; depression, anxiety, or psychological distress; sensation seeking; parental monitoring; perceived risk of harm; outcome expectancies, defined as the believed consequences of a person’s behavior; past use of illicit drugs; criminal history; and body mass index (BMI) (6–11).

Despite studies establishing the factors affecting marijuana use, few studies have used a framework that could model multiple pathways and mediating effects. Structural equation modeling comprehensively analyzes complex relationships between observed and unobserved variables by including direct and indirect effects (12) and is therefore appropriate for exploring the complex pathways of marijuana use among women aged 18 to 44 years.

This study examined the potential predictors and mediators of marijuana use among US women aged 18 to 44 years.

## Methods

### Data source

This study used data from the 2023 NSDUH. The NSDUH provides nationally representative data from a civilian, noninstitutionalized population aged 12 years or older in the US on the use of tobacco, alcohol, and other drugs; substance use disorders; mental health issues; and receipt of substance use and mental health treatment (1). These data are publicly available.

### Study sample

The sample was restricted to women aged 18 to 44 years who reported past-year marijuana use. After excluding those with missing variables, the final sample size was 16,081.

### Measures

#### Outcome variable

The dependent variable was past-year marijuana use. Past-year marijuana use was a self-reported variable in which participants were asked whether they used marijuana in the past year (yes/no) and was coded as a binary variable.

#### Predictor variables

Age was reported in years and analyzed as a categorical variable (18–25, 26–34, 35–44 y). Race and ethnicity was categorized as Hispanic, non-Hispanic Asian, non-Hispanic American Indian/Alaska Native, non-Hispanic Black/African American, non-Hispanic Native Hawaiian/Pacific Islander, non-Hispanic White, and more than 1 race. Marital status was a categorical variable coded as never married (widowed or divorced), currently married, or formerly married. Education was categorized as less than high school graduate, high school graduate, some college or an associate’s degree, or college graduate or higher. County type, a categorical variable, was classified based on the 2013 Rural-Urban Continuum Codes (13) as large metropolitan, small metropolitan, and nonmetropolitan. We also included binge drinking (no/yes); past criminal history was a binary variable categorized as respondents having been arrested and booked for breaking the law in the past 12 months (no/yes), excluding minor traffic offenses.

#### Mediator variables

The past-year major depressive episodes (PY–MDE) variable was defined as having an MDE in the prior year. Past-year use of illicit drugs, tobacco, and alcohol (PY–IDTA) other than marijuana was a composite binary variable created by using the past-year use of illicit drugs, tobacco, and alcohol other than marijuana variables. Poverty status was categorized based on federal poverty thresholds (ie, living in poverty, income up to 2 times the federal poverty threshold, and income more than 2 times the federal poverty threshold) (14), and BMI was a categorical variable (under, normal, over, and obese) constructed from the recorded BMI values.

### Statistical analysis

We used descriptive statistics (frequencies and weighted percentages) to summarize sample sociodemographic variables (age, race and ethnicity, marital status, education, poverty status, county type).

We employed structural equation modeling to examine the direct and indirect pathways of exogenous (ie, observed) and endogenous (ie, latent) variables. To examine the direct and indirect relationship between the sociodemographic variables and past-year marijuana use, we conducted structural equation modeling. We assessed model fit using standard goodness-of-fit indices: comparative fit index (CFI ≥0.90), Tucker–Lewis index (TLI ≥0.90; root mean square error of approximation (RMSEA ≤0.06), and standardized root mean square residual (SRMR ≤ 0.08) (15). We also calculated 95% CIs using bias-corrected bootstrap SEs (16). We included diagrammatic description of all direct and indirect pathways for 1) all pathways regardless of their significance and 2) only significant pathways. Sampling weights were applied to the analyses to account for the complex survey design. All analyses were conducted using Mplus version 8.11 (Muthén and Muthén). Listwise deletion was used to handle missing data and ensure its completeness. We applied WLSMV (weighted least squares mean and variance adjusted) with THETA (Theta parameterization).

## Results

Of the total study sample (N = 16,081), 30.3% (n = 5,294) of women reported past-year marijuana use. Of the total sample, 37.5% (n = 4,630) were aged 35 to 44 years, 54.7% (n = 8,772) were non-Hispanic White, 51.3% (n = 9,203) were never married, and 38.0% (n = 5,539) were college graduates or higher. Moreover, 56.2% (n = 7,145) were residents from large metropolitan areas, 16.1% (n = 2,714) reported binge drinking in the past month, 9.5% (n = 1,486) reported a past criminal history and reported experiencing MDE in the past year, 28.8% (n = 4,553). Additionally, 11.1% (n = 1,851) reported using illicit drugs other than marijuana, 59.1% (n = 8,850) reported having income more than 2 times the federal poverty threshold, and 37.1% (n = 5,834) had obesity.

From the bivariate analyses, by sociodemographic characteristics, we found that a significantly larger proportion of women who used marijuana in the past year were in the following groups: those aged 26 to 34 years (χ^2^ = 171.0, *P* < .001), those who were non-Hispanic White (χ^2^ = 340.3, *P* < .001), those who were never married (χ^2^ = 486.0, *P* < .001); those who had some college or an associate’s degree (χ^2^ = 91.0, *P* < .001); those who reported having no MDE (χ^2^ = 434.5, *P* < .001), not binge drinking (χ^2^ = 1,029.9, *P* < .001), or not having PY–IDTA (χ^2^ = 1,717.6, *P* < .001); and those without a past criminal history (χ^2^ = 384.0, *P* < .001) (Table 1).

**Table 1:** Bivariate Analysis With the Outcomes of Marijuana Use, Women Aged 18–44 Years Who Reported Past-Year Marijuana Use, 2023 National Drug Use and Health Survey

Characteristic	Marijuana use, no. (%^a^)			χ**^2^** (*P* value)
	No. (n = 10,787)	Yes (n = 5,294)	Total (n = 16,081)	
**Age, y**				
18–25	4,150 (26.0)	2,499 (33.9)	6,649 (28.4)	171.0 (<.001)
26–34	3,257 (33.4)	1,545 (35.6)	4,802 (34.1)	
35–44	3,380 (40.6)	1,250 (30.5)	4,630 (37.5)	
**Race and ethnicity**				
Hispanic	2,491 (22.7)	956 (16.9)	3,447 (20.9)	340.3 (<.001)
Non-Hispanic American Indian/Alaska Native	134 (0.4)	102 (0.7)	236 (0.5)	
Non-Hispanic Asian	679 (9.3)	124 (3.0)	803 (7.4)	
Non-Hispanic Black	1,311 (13.4)	631 (14.3)	1,942 (13.7)	
Non-Hispanic Native Hawaiian/Pacific Islander	53 (0.3)	20 (0.5)	73 (0.4)	
Non-Hispanic White	5,657 (52.0)	3,115 (60.8)	8,772 (54.7)	
More than 1 race	462 (2.0)	346 (3.8)	808 (2.5)	
**Marital status**				
Married	4,304 (44.4)	1,262 (26.0)	5,566 (38.9)	486.0 (<.001)
Widowed	47 (0.6)	27 (0.6)	74 (0.6)	
Divorced	793 (8.6)	445 (10.9)	1,238 (9.3)	
Never married	5,643 (46.4)	3,560 (62.5)	9,203 (51.3)	
**Education**				
Less than high school	1,053 (7.1)	457 (5.4)	1,510 (6.56)	91.0 (<.001)
High school graduate	2,666 (22.2)	1,342 (22.4)	4,008 (22.3)	
Some college/associate’s degree	3,173 (31.1)	1,851 (38.1)	5,024 (33.2)	
College graduate or higher	3,895 (39.6)	1,644 (34.1)	5,539 (38.0)	
**County**				
Large metropolitan	4,778 (56.0)	2,367 (56.7)	7,145 (56.2)	5.6 (.24)
Small metropolitan	4,272 (31.5)	2,070 (32.2)	6,342 (31.7)	
Nonmetropolitan	1,737 (12.5)	857 (11.2)	2,594 (12.1)	
**Past-month binge drinking**				
No	8,592 (78.8)	2,936 (53.8)	11,528 (71.2)	1,029.9 (<.001)
Yes	2,195 (21.2)	2,358 (46.2)	4,553 (28.8)	
**Past criminal history**				
No	10,120 (93.5)	4,475 (83.7)	14,595 (90.5)	384.0 (<.001)
Yes	667 (6.5)	819 (16.3)	1,486 (9.5)	
**Past-year major depressive episodes**				
No	9,434 (87.9)	3,933 (74.8)	13,367 (83.9)	434.5 (<.001)
Yes	1,353 (12.1)	1,361 (25.2)	2,714 (16.1)	
**Past year illicit drug, tobacco, and alcohol use**				
No	10,327 (95.7)	3,903 (73.4)	14,230 (88.9)	1,717.6 (<.001)
Yes	460 (4.31)	1,391 (26.6)	1,851 (11.1)	
**Poverty status**				
Living in poverty	2,583 (20.7)	1,353 (21.4)	3,936 (20.9)	1.3 (.70)
Income 2 times federal poverty threshold	2,185 (19.9)	1,110 (20.2)	3,295 (20.0)	
Income more than 2 times federal poverty threshold	6,019 (59.4)	2,831 (58.5)	8,850 (59.1)	
**Body mass index**				
Underweight	343 (3.3)	194 (3.4)	537 (3.3)	0.8 (>.99)
Normal weight	3,922 (35.2)	1,920 (34.7)	5,842 (35.0)	
Overweight	2,617 (24.7)	1,251 (24.4)	3,868 (24.6)	
Obese	3,905 (36.9)	1,929 (37.5)	5,834 (37.1)	

a Sampling weights were applied to the analyses to account for the complex survey design. Percentages may not add to 100 because of rounding.

Model fit statistics indicated the following: χ^2^_6_ = 116.4, *P* < .001; RMSEA = 0.034; CFI = 0.941; TLI = 0.556; SRMR = 0.028; χ^2^_45_ = 1,908.5, *P* < .001 (Table 2). According to the structural equation model, past criminal history (β = 0.188), binge drinking (β = 0.404), PY–MDE (β = 0.310), and PY–IDTA (β = 0.658) were positively associated with marijuana use, while being younger in age (β = -0.065, *P* = .007) was associated with an increase in marijuana use (Figure). In the model containing only significant pathways (Table 3), age had a negative direct effect (β = −0.065, *P* = .007) and a negative indirect effect in the presence of MDE (β = −0.040, *P* < .001) on past-year marijuana use. Race/ethnicity showed a negative direct effect (β = −0.027, *P* < .001) and negative indirect effects in the presence of MDE (β = −0.008, *P* < .001) and PY–IDTA (β = −0.013, *P* = .002) on marijuana use. Similarly, marital status had a positive direct effect in the presence of MDE (β = 0.030, *P* < .001) and PY–IDTA (β = 0.076, *P* < .001) on marijuana use. Education had a positive direct effect on past-year marijuana use (β = 0.042, *P* = .003) if the respondents had PY–IDTA use. Likewise, county had a positive indirect effect on past-year marijuana use (β = 0.013, *P* =0.036) if the respondents had reported PY–MDE. Additionally, those who reported a past criminal history showed a significant positive direct effect (β = 0.188, *P* = .003) and an indirect effect through PY–MDE (β = 0.080, *P* < .001) and PY–IDTA (β = 0.378, *P* < .001) on marijuana use. Binge drinking also had a positive direct effect (β = 0.404, *P* < .001) and a positive indirect effect through PY–IDTA (β = 0.238, *P* < .001) on marijuana use. 

**Table 2:** Model Fit Statistics for the Structural Equation Model Examining Marijuana Use Among US Women Aged 18–44 Years Who Reported Past-Year Marijuana Use, 2023 National Drug Use and Health Survey

Fit index	Value	Interpretation
Model χ^2^	χ^2^_6_ = 116.4 (*P* < .001)	Not a good fit
Root mean square error approximation	0.034	Good fit
Comparative fit index	0.941	Acceptable fit
Tucker–Lewis index	0.556	Not a good fit
Standardized root mean square residual	0.028	Good fit
Base model χ^2^	χ^2^_45_ = 1,908.5 (*P* < .001)	Significant

**Figure:**
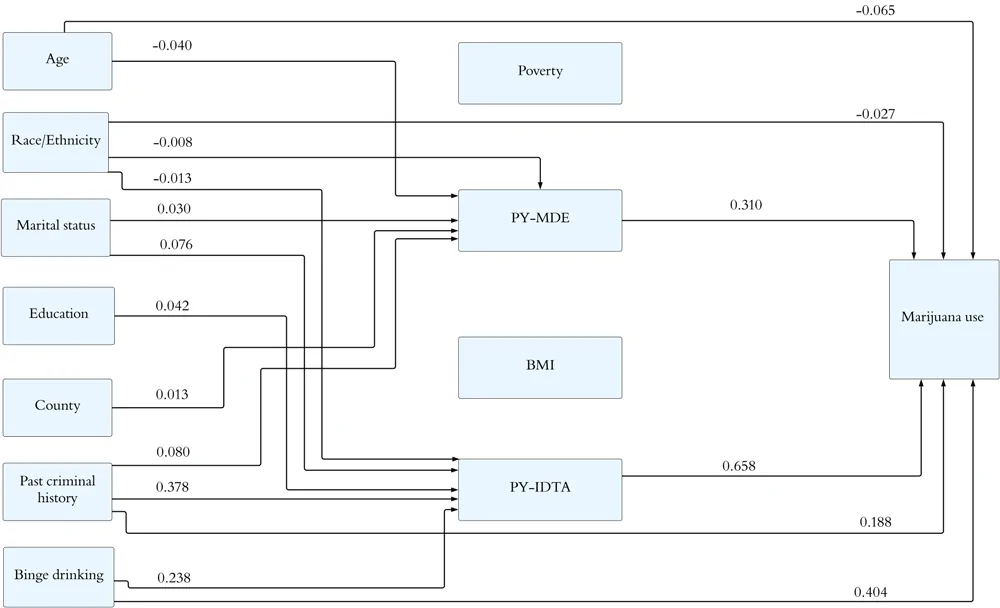
Structural equation model showing significant pathways associated with marijuana use among US women aged 18 to 44 years, 2023 National Survey on Drug Use and Health. Values shown are standardized pathway coefficients (β). Only significant pathways (*P* < .05) are displayed. Abbreviations: BMI, body mass index; PY–MDE, past year major depressive episode; PY–IDTA, past year illicit drug, tobacco, and alcohol use.

**Table 3:** Total, Direct, and Indirect Effects From the Structural Equation Model, Women Aged 18–44 Years Who Reported Past-Year Marijuana Use, 2023 National Drug Use and Health Survey

Effect	Point estimate (95% CI)	SE	*P* value
**Effect of age on marijuana use**			
Total	−0.106 (−0.155 to −0.077)	0.028	<.001
Total indirect	−0.041 (−0.085 to −0.012)	0.023	.03
**Specific indirect**			
Age–poverty status–marijuana use	0.001 (0.000 to 0.004)	0.001	.48
Age–body mass index–marijuana use	0.008 (−0.004 to 0.018)	0.007	.18
Age–PY–IDTA–marijuana use	−0.010 (−0.044 to 0.023)	0.021	.57
Age–MDE–marijuana use	−0.040 (−0.060 to −0.028)	0.009	<.001
Age–marijuana use	−0.065 (−0.124 to −0.027)	0.030	.01
**Effects of race on marijuana use**			
Total	−0.049 (−0.062 to −0.039)	0.008	<.001
Total indirect	−0.022 (−0.032 to −0.009)	0.006	<.001
Race–poverty status–marijuana use	−0.001 (−0.003 to 0.001)	0.001	.39
Race–body mass index–marijuana use	0.000 (0.000 to 0.001)	0.000	.30
Race–PY–IDTA–marijuana use	−0.013 (−0.022 to −0.005)	0.005	<.002
Race–MDE–marijuana use	−0.008 (−0.013 to −0.003)	0.003	<.001
Race–marijuana use	−0.027 (-0.043 to −0.018)	0.008	<.001
**Effects of marital status on marijuana use**			
Total	0.134 (0.105 to 0.160)	0.017	<.001
Total indirect	0.105 (0.076 to 0.124)	0.015	<.001
Marital status–poverty status–marijuana use	−0.003 (−0.008 to 0.003)	0.004	.38
Marital status–body mass index–marijuana use	0.001 (−0.001 to 0.003)	0.001	.20
Marital status–PY–IDTA–marijuana use	0.076 (0.055 to 0.091)	0.012	<.001
Marital status–PY–MDE–marijuana use	0.030 (0.022 to 0.038)	0.005	<.001
Marital status–marijuana use	0.029 (0.002 to 0.058)	0.019	.06
**Effects on education on marijuana use**			
Total	0.021 (−0.017 to 0.063)	0.023	.25
Total indirect	0.052 (0.020 to 0.078)	0.019	<.001
Education–poverty status–marijuana use	0.009 (−0.011 to 0.029)	0.012	.38
Education–body mass index–marijuana use	−0.003 (−0.008 to 0.001)	0.003	.18
Education–PY–IDTA–marijuana use	0.042 (0.011 to 0.072)	0.018	<.001
Education–PY–MDE–marijuana use	0.004 (−0.008 to 0.014)	0.007	.48
Education–marijuana use	−0.031 (−0.080 to 0.007	0.026	.14
**Effects of county on marijuana use**			
Total	−0.060 (−0.102 to −0.028)	0.029	.01
Total indirect	−0.018 (−0.076 to 0.021)	0.026	.39
County–poverty status–marijuana use	−0.003 (−0.01 to 0.004)	0.005	.39
County–body mass index–marijuana use	0.003 (−0.002 to 0.008)	0.003	.20
County–PY–IDTA–marijuana use	−0.031 (−0.067 to 0.008)	0.024	.10
County–PY–MDE–marijuana use	0.013 (−0.001 to 0.026)	0.008	.04
County–marijuana use	−0.042 (−0.127 to 0.000)	0.037	.16
**Effects of past criminal history on marijuana use**			
Total	0.642 (0.544 to 0.741)	0.075	<.001
Total indirect	0.454 (0.374 to 0.560)	0.060	<.001
Past criminal history–poverty status–marijuana use	−0.004 (−0.017 to 0.004)	0.007	.42
Past criminal history–body mass index–marijuana use	0.001 (−0.001 to 0.007)	0.002	.61
Past criminal history–PY–IDTA–marijuana use	0.378 (0.310 to 0.456)	0.050	<.001
Past criminal history–PY–MDE–marijuana use	0.080 (0.044 to 0.115)	0.020	<.001
Past criminal history–marijuana use	0.188 (0.079 to 0.311)	0.077	<.001
**Effects of binge drinking on marijuana use**			
Total	0.659 (0.568 to 0.726)	0.049	<.001
Total indirect	0.254 (0.219 to 0.31)	0.036	<.001
Binge drinking–poverty status–marijuana use	0.002 (−0.002 to 0.008)	0.003	.38
Binge drinking–body mass index–marijuana use	0.000 (−0.003 to 0.002)	0.001	.92
Binge drinking–PY–IDTA–marijuana use	0.238 (0.197 to 0.285)	0.035	<.001
Binge drinking–PY–MDE–marijuana use	0.014 (−0.014 to 0.029)	0.012	.15
Binge drinking–marijuana use	0.404 (0.301 to 0.475)	0.047	<.001

Abbreviations: PY–IDTA, past year illicit drug, tobacco, and alcohol use; PY–MDE, past year major depressive episodes.

## Discussion

Our study applied structural equation modeling to analyze data from the 2023 NSDUH to examine the potential predictors and mediators of marijuana use among US women aged 18 to 44 years. Our findings suggest that about 30% of women of reproductive age reported marijuana use in the past year. However, this percentage is higher than the percentage found in a prior study using 2021–2023 NSDUH pooled data in which the prevalence of marijuana use was about 12.6% among nonpregnant women (17). The discrepancy suggests the need for further investigation; the rising prevalence underscores the importance of understanding the personal, behavioral, and environmental factors that may affect marijuana use in women.

Our study identified direct and indirect pathways linking sociodemographic factors, poverty status, PY–MDE, PY–IDTA, BMI, and past-year marijuana use among women. From the structural equation modeling analysis, both age and race and ethnicity had a negative direct effect on the use of marijuana. Younger age was associated with a higher likelihood of marijuana use, consistent with previous studies documenting sociodemographic differences in marijuana use (18). For younger people, factors such as social isolation, peer pressure, stress, poor grades, family issues, and curiosity may account for the use of marijuana (19). Those in racial and ethnic minority groups had higher levels of marijuana use, consistent with previous research; previous research demonstrated that marijuana use was driven by several factors unique to these demographic characteristics, such as cultural stress and environmental stressors (20). Past criminal history and binge drinking also had positive direct effects on marijuana use, findings supported by previous studies showing marijuana use in those with a history of criminal justice involvement, alcohol use, and polysubstance use (21–23).

A notable contribution of our study is that PY–MDE and PY–IDTA acted as mediators of marijuana use, highlighting the connection between psychological distress and polysubstance use and marijuana use. PY–MDE was identified as mediating age, past criminal history, binge drinking, and marijuana use. Sociodemographic characteristics do not act separately in the vulnerability to marijuana use. These findings are similar to those in a previous study that found sociodemographic factors to be associated with mental health outcomes (24). Depressive episodes may increase vulnerability to substance use (25); substance use may act as a coping mechanism for distress (26). Addressing MDE can therefore influence the use of marijuana. Limiting marijuana use can be beneficial to health in the long term; for example, limiting use can reduce peripheral vascular diseases (27).

PY–IDTA had even larger indirect effects than PY–MDE, mediating race and ethnicity, marital status, education, criminal history, binge drinking, and marijuana use, consistent with pathways seen in other studies (28). This finding underscores the consequences of substance use vulnerability, where using one substance may lead to the use of another, and aligns with studies showing that marijuana is most often used in the presence of other illicit drugs (29).

Identifying these mediating factors is important to highlight the need for prevention programs to focus on addressing psychological and polysubstance predictors of use rather than solely on marijuana use.

### Strengths and limitations

Strengths of this study are the use of structural equation modeling to examine direct and indirect pathways of marijuana use and the use of a nationally representative sample. Our findings should be interpreted with consideration of some limitations. Because our study was cross-sectional, we cannot make causal inferences. Additionally, the data were based on a self-reported questionnaire, which may introduce recall and social desirability biases. Although our model had a good fit on numerous indices, the χ^2^ test of the model failed to reject the null hypothesis, and TLI was below the recommended value. However, the χ^2^ test of a model can be significant if the sample is large, which is the case with our model (30). Similarly, low TLI with a good fit of other indices is not uncommon (31). In our study, indices other than TLI were of good fit (eg, RMSEA, SRMR, CFI), supporting the hypothesized pathways.

### Implications

Implications of our study include the identification of gender-based approaches to address predictors of marijuana use among women aged 18 to 44 and limit its use in this population. By identifying the various pathways for marijuana use among women, interventions can be implemented at time points identified as most effective for women. Additionally, our study shows the connections in women among marijuana use, mental health struggles, and conditions like depression and the use of other substances, which suggests an opportunity to strengthen referrals in the health care system. Efficient referral and screening systems could support primary care providers in improving timely screening uptake among women and promoting early intervention.

Further research into the predictors of marijuana use among women, particularly those of reproductive age, is necessary. Longitudinal studies measuring long-term predictors of marijuana use and its effects could add to the current understanding of how marijuana affects health over time. Marijuana use among women continues to be an underresearched area (8), and it may have consequences that cannot be mitigated without further research. Additional research could explore marijuana-specific policies and how they influence the pathways identified in our study. Finally, qualitative studies examining the experiences of women who use marijuana can aid in supporting measures to mediate predictors and give more insight into how these complex influences connect and directly affect use.

### Conclusion

Overall, our study demonstrates that marijuana use among US women is affected by an interplay of sociodemographic, psychological, and behavioral factors, with depression and use of other illicit drugs being key mediators. Prevention efforts should move away from addressing single factors related to marijuana use among women and instead use multiple approaches targeting stress and polysubstance use in this group.
